# The impact of COVID-19 on gastric cancer surgery: a single-center retrospective study

**DOI:** 10.1186/s12893-020-00885-7

**Published:** 2020-10-02

**Authors:** Yu-xuan Li, Chang-zheng He, Yi-chen Liu, Peng-yue Zhao, Xiao-lei Xu, Yu-feng Wang, Shao-you Xia, Xiao-hui Du

**Affiliations:** 1 Department of General Surgery, Chinese PLA General Hospital, Beijing, 100853 PR China; 2 Department of Hospitalization management, Chinese PLA General Hospital, Beijing, 100853 PR China

**Keywords:** Gastric cancer, Coronavirus disease 2019, COVID-19, Retrospective analysis

## Abstract

**Background:**

The coronavirus disease 2019 (COVID-19) has been declared a global pandemic by the World Health Organization. Patients with cancer are more likely to incur poor clinical outcomes. Due to the prevailing pandemic, we propose some surgical strategies for gastric cancer patients.

**Methods:**

The ‘COVID-19’ period was defined as occurring between 2020 and 01-20 and 2020-03-20. The enrolled patients were divided into two groups, pre-COVID-19 group (PCG) and COVID-19 group (CG). A total of 109 patients with gastric cancer were enrolled in this study.

**Results:**

The waiting time before admission increased by 4 days in the CG (PCG: 4.5 [IQR: 2, 7.8] vs. CG: 8.0 [IQR: 2,20]; *p* = 0.006). More patients had performed chest CT scans besides abdominal CT before admission during the COVID-19 period (PCG: 22 [32%] vs. CG: 30 [73%], *p* = 0.001). After admission during the COVID period, the waiting time before surgery was longer (PCG: 3[IQR: 2,5] vs. CG: 7[IQR: 5,9]; *p* < 0.001), more laparoscopic surgeries were performed (PCG: 51[75%] vs. CG: 38[92%], *p* = 0.021), and hospital stay period after surgery was longer (7[IQR: 6,8] vs.9[IQR:7,11]; *p* < 0.001). In addition, the total cost of hospitalization increased during this period, (PCG: 9.22[IQR:7.82,10.97] vs. CG: 10.42[IQR:8.99,12.57]; *p* = 0.006).

**Conclusion:**

This study provides an opportunity for our surgical colleagues to reflect on their own services and any contingency plans they may have to tackle the COVID-19 crisis.

## Background

A respiratory epidemic defined as ‘coronavirus disease 2019 (COVID-19)’ emerged in Wuhan city, Hubei Province, China. Forty-one patients were admitted to hospitals with an initial pneumonia diagnosis of an unknown etiology. Most of these patients had visited a local fish and wild animal market in November [1, 2]. Since then, COVID-19 has been declared a global pandemic by the World Health Organization (WHO). According to the situation report-77 of WHO (Data as of 6th April, 2020), more than 1.2 million people had been infected across the globe [3].

Most patients presented with fever, dry cough and dyspnea. However, incidences of isolated or coexisting abdominal and gastrointestinal symptoms such as diarrhea, nausea, vomiting and abdominal discomfort were also common [4]. Due to the route of transmission of this disease, stringent precautionary measures for patients and particularly hospital staffs who were at great risk were implemented [5, 6]. Through droplet and contact transmission, the virus can be spread by asymptomatic patients. The safety of nurses, surgeons, patients and their families is of paramount importance [7, 8].

Gastric cancer surgery is not a front-line subject in the fight against COVID-19, however, in such a special situation, due to disease consumption, malnutrition, coupled with chemotherapy, gastric cancer patients may be immunocompromised, which leads to more susceptible to COVID-19 and poor clinical outcomes [9, 10]. Based on our experiences during the pandemic period, we propose some surgical strategies for gastric cancer patients.

## Methods

### Study design and patients

The General Surgery Department of our hospital serves as a final referral unit for a cluster of hospitals from other districts and provides specialized services for gastric cancer. On January 20th, 2020, the National Health Commission announced that the prevention and control measures for the COVID-19 infectious would be category A management. As a result, intensified clinical management strategies for outpatients, inpatients and discharged COVID-19 cases were intensified. On March 20th 2020, and the consecutive days, there were no new locally confirmed COVID-19 cases in Beijing. This was a milestone in the battle against this virus. Based on the above factor, we defined the ‘COVID-19’ period as occurring between 2020 and 01-20 and 2020-03-20. Data obtained during this period was compared with a similar preceding 1-month period between 2020 and 12-20 and 2020-1-19 which we termed the ‘Pre-COVID-19’ period. Determined by which period the enrolled patients were admitted to the hospital, they were divided into two groups; the pre-COVID-19 group (PCG) and the COVID-19 group (CG).

We aimed to compare the differences in demographics, baseline characteristics, clinicopathological features, and health economics between the two groups to investigate the feasibility of gastric surgery during the pandemic of COVID-19.

This study was approved by the Institutional Review Board of the General Hospital of PLA. Inclusion criteria were: i. Patients diagnosed with gastric cancer by pathological examinations and whose electronic medical records were available; ii. Patients who received surgical treatment. On the contrary, patients who received neoadjuvant chemotherapy (NACT) or had emergency surgical procedures were excluded. A total of 109 patients with gastric cancer were enrolled in this study. They were diagnosed according to The NCCN Clinical Practice Guidelines in Oncology (NCCN Guidelines) for Gastric Cancer. Operations were performed by the same team of surgeons.

### Data collection

The medical records of patients were obtained and analyzed by our research team. The clinical, epidemiological, radiological, laboratory characteristics from electronic medical records were summarized. These data included patient demographics and baseline characteristics (sex, age, body mass index, comorbidity, clinical TNM classification, pathological TNM classification, hemoglobin, CEA, CA199, AFP, CA724), origin of patients (from local district or other provinces), operative method (open surgery or laparoscopic surgery), operating time, estimated blood loss, postoperative complications, postoperative fever, waiting time before admission, length of postoperative hospital stay, hospital costs etc. We defined the waiting time before admission as the period from the time when the patient came to our outpatient clinic to hospitalization. Length of postoperative hospital stay was defined as the period from the time when patient had undergone surgery to discharge.

### Statistical analysis

The SPSS version 26.0 was used for statistical analysis. The assumption of data normality for all quantitative variables data was verified with the Shapiro-Wilk test. For the normally distributed variables, data were expressed as mean ± standard deviation(푥̅±s). The median and interquartile ranges were used to express measurement data that did not conform to normal distribution. Count data were expressed by frequency and percentage (%). The students t test was used to compare the means for normally distributed variables while the Mann-Whitney U test was used for variables without normal distribution. Statistical analysis of count data was done using chi-square test or Fisher exact probability method. The *p*-value less than 0.05 was considered significant.

## Results

### Study population and baseline demographics

**Fig. 1:**
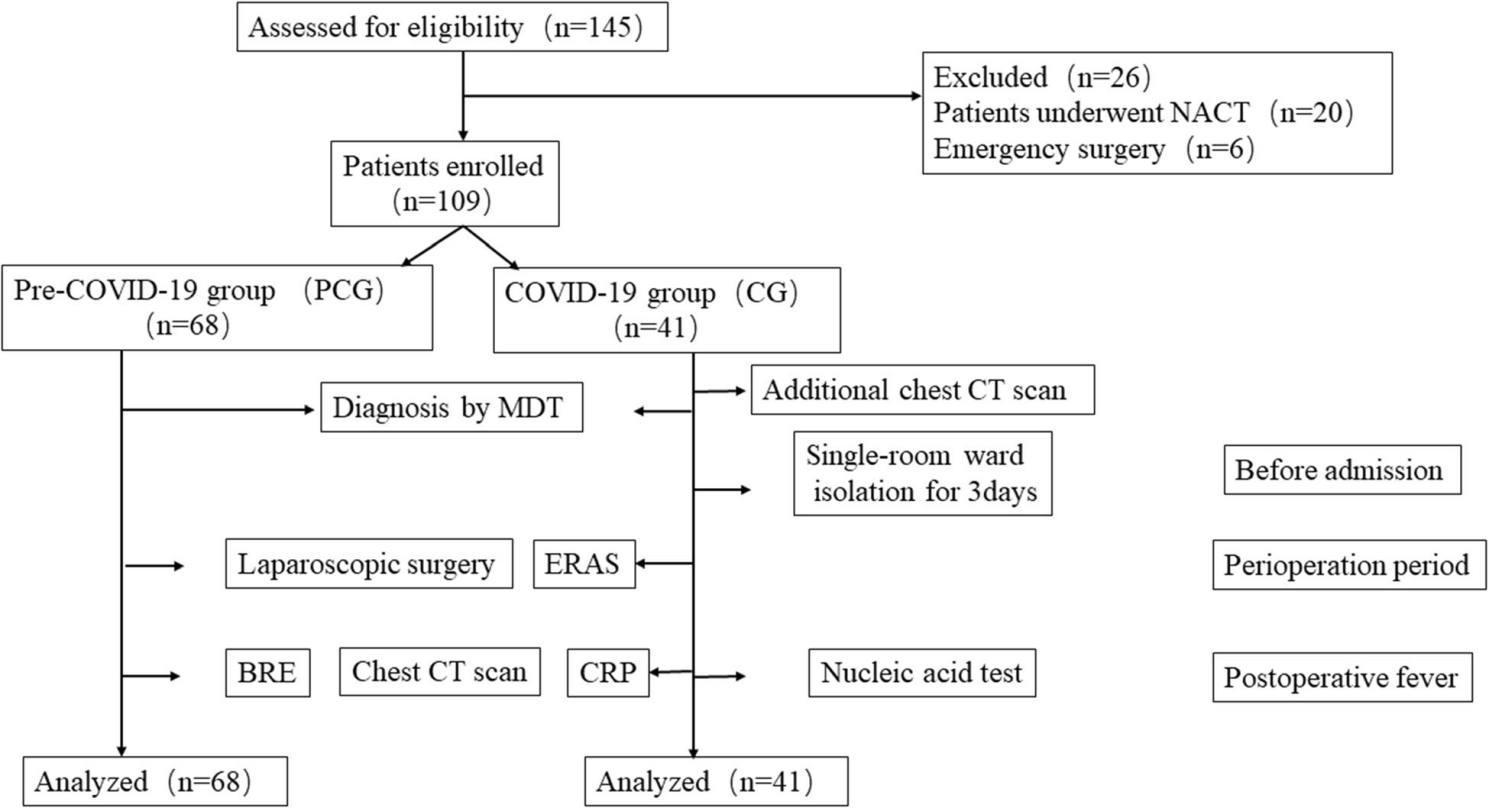
Flow diagram of screening and enrollment

Between December 20th, 2019 and March 20th, 2020, a total of 109 patients were enrolled. In this study, 68 were enrolled into PCG while 41 were enrolled into CG (Fig. 1). Patient demographics for PCG and CG were shown in Table 1. There was no statistically significant difference in age, sex and body mass index between the two groups (*p* > 0.05).

**Table 1:** Baseline demographical data of all patients enrolled

Demographics	PCG (*N* = 68) M ± SD or N (%)	CG (*N* = 41) M ± SD or N (%)	*P*
Age (years)	59.60 ± 11.04	58.41 ± 10.27	0.578
Sex			0.583
Male	45	25	
Female	23	16	
BMI	24.37 ± 3.68	23.30 ± 2.91	0.099
Origin of patients			0.000*
Local patients	3	16	
Other provinces	65	25	

*PCG* Pre-COVID-19 group, *CG* COVID-19 group, *M* Mean, *SD* Standard deviation, *N* Number

* *P* < 0.05, statistically different

### Clinicopathological data of all patients enrolled before or after 20th January

Pre-COVID-19, admissions to the gastric unit totaled 68 cases over the 1-month period. During COVID-19, this total dropped by 30% to 41 admissions. In contrast, the waiting time before admission increased by 4 days (PCG:4.5 [IQR: 2, 7.8] vs. CG:8.0 [IQR: 2,20]; *p* = 0.006). The proportion of local patients in PCG was lower when compared to those in CG (3 [4%] vs. 16 [39%]; *p* < 0.001). More patients had performed chest CT scan besides abdominal CT before admission during the COVID-19 period (PCG:22[32%] vs. CG:30[73%], *p* = 0.001). These results were shown in Table 2.

**Table 2:** Preoperative clinicopathological data of all patients enrolled

Clinicopathologic data	PCG(*N* = 68) M (IQR) orN (%)	CG (*N* = 41) M (IQR) orN (%)	*P*
Admission waiting (day)	4.5 (2–7.75)	8.0 (2–20)	0.006*
Operation waiting (day)	3 (2–5)	7 (5–9)	0.000*
Comorbidity			0.144
Yes	46	22	
No	22	19	
Tumor marker			0.298
CEA	2.12	1.85	0.771
AFP	2.85	3.01	0.883
CA199	9.70	9.63	0.863
CA724	2.03	2.13	
Clinical TNM stage			0.112
I	17	4	
II	18	14	
III	30	23	
IV	3	0	
Hemoglobin	138.50 (119.25–148.75)	143.00 (119.50–151.00)	0.726
Chest CT scan			0.000*
Yes	24	30	
No	44	11	

*PCG* Pre-COVID-19 group, *CG* COVID-19 group, *CT* Computed tomography; c, *N* Number

* *P* < 0.05, statistically different

During the COVID-19 period, the waiting time before surgery was longer (PCG: 3[IQR: 2,5] vs. CG: 7[IQR: 5,9]; *p* < 0.001) with more laparoscopic surgeries being performed (PCG: 51[75%] vs. CG: 38[92%], *p* = 0.021). In addition, the length of hospital stay after surgery was longer (7[IQR: 6,8] vs.9[IQR:7,11]; p < 0.001). There were no significant statistical differences in surgical time, pathological diagnosis, TNM staging, and in complications associated with pneumonia, blood transfusion, the highest temperature and screening test between the two groups (*p* > 0.05). These results were shown in Tables 3 and 4.

**Table 3:** Intraoperative clinicopathological data of all patients enrolled

Clinicopathologic data	PCG (*N* = 68) M ± SD or N (%)	CG (*N* = 41) M ± SD or N (%)	*P*
Surgery time (min)	184.74 ± 44.36	195.00 ± 57.49	0.298
Estimated blood loss (ml)^a^	100 (50–137)	100 (50–150)	0.569
Operative method			0.021*
Open	17	3	
Laparoscopic	51	38	
Combine organ resection			0.000*
Yes	1	0	
No	67	41	
Transfusion of blood			0.172
Yes	4	6	
No	64	35	

*PCG* Pre-COVID-19 group, *CG* COVID-19 group, *M* Mean, *SD* Standard deviation, *N* Number. ^a^*M* Median, *IQR* Inter-quartile range

* *P* < 0.05, statistically different

**Table 4:** Postoperative clinicopathological data of all patients enrolled

Clinicopathologic data	PCG(*N* = 68) M (IQR) orN (%)	CG (*N* = 41) M (IQR) orN (%)	*P*
Pathological TNM staging			0.394
I	18	8	
II	18	10	
III	29	23	
IV	3	0	
Complications			0.081
Yes	6	0	
No	62	41	
Postoperative fever			0.379
Yes	29	14	
No	39	27	
Highest temperature (°C)			0.587
< 37.3	39	27	
37.3–38.5	22	12	
> 38.5	7	2	
Screening examination			0.012*
None	16	2	
BRE + CRP	8	4	
BRE + CRP + CT	4	5	
Nucleic acid test	0	2	
Reason of fever			0.423
Abdominal infection	1	0	
Pulmonary infection	1	0	
Incision infection	4	0	
Anastomotic leakage	1	0	
Unclear	17	11	
None	5	3	
Transfusion of blood			0.422
Yes	3	4	
No	65	37	
Postoperative hospital stay (day)	7 (6–8)	9 (7–11)	0.000*
Total hospital stay (day)	11 (9–13)	15 (13–19)	0.000*

*PCG* Pre-COVID-19 group, *CG* COVID-19 group, *BRE* Blood routine examination, *CRP* C-reactive protein, *CT* Computed tomography, Postoperative fever (≥37.3 °C)

*M* Median, *IQR* Inter-quartile range, *N* Number

* *P* < 0.05, statistically different

### Health economics data of all patients enrolled before or after 20th January

The total cost of hospitalization increased during the COVID-19 period, (PCG: 9.22[IQR:7.82,10.97] vs. CG: 10.42[IQR:8.99,12.57]; *p* = 0.006), The cost of medicines, treatment and other aspects such as beds, diets etc. were higher in CG (p>0.05). These results were shown in Table 5.

**Table 5:** Health economics data of all patients enrolled

Health economics data (10,000 RMB)	PCG(*N* = 68) M (IQR)	CG (*N* = 41) M (IQR)	*P*
Medicine	2.51 (1.79–3.27)	2.89 (2.29–4.30)	0.002*
Examination	0.05 (0.03–0.09)	0.06 (0.05–0.08)	0.051
Laboratory test	0.83 (0.68–1.06)	1.00 (0.76–1.18)	0.078
Treatment	7.99 (6.59–9.66)	9.00 (7.59–11.09)	0.024*
Surgery	0.46 (0.45–0.47)	0.47 (0.40–0.47)	0.527
Anesthesia	0.20 (0.18–0.23)	0.22 (0.19–0.23)	0.131
Consumables	3.94 (3.55–4.50)	4.45 (3.45–5.45)	0.140
Others	0.26 (0.21–0.34)	0.47 (0.36–0.54)	0.000*
Total costs	9.23 (7.82–10.97)	10.42 (8.99–12.57)	0.006*

*PCG* Pre-COVID-19 group, *CG* COVID-19 group, *M* Median, *IQR* Inter-quartile range

* *P* < 0.05, statistically different

## Discussion

In this study, the 30% fall in case load during the COVID-19 period and the proportion of local patients in PCG being lower than those in CG could be attributed to the travel restrictions and lockdown imposed on Beijing. During the COVID-19 period, the use of telemedicine and remote counselling has made great strides. This has helped in reducing the number of outpatients and unnecessary physical contacts. Tolone et al. used triage questionnaires for elective surgical patients in cases of positive symptoms and contact history associated with COVID-19. These questionnaires were administered through the telephone [11]. Gambardella et al. reported their experience regarding treatment for old cancer patients. They documented several procedures that could help in preventing disease transmission among patients. These procedures encompassed the use of a telephone triage before admission, and the application of telemedicine [12]. In CG, appointments and triage protocols were to be performed virtually through telemedicine such as mobile phones, applications or the websites, thereby, clinical visits were to be performed based on reserved numbers and recommended time.

The COVID-19 outbreak brought to the importance of infection control measures for pandemic diseases. Successful implementation of infection control measures require the strict management of inpatients during this period. Patients with cancers have been established to be immunocompromised, which makes them more susceptible to COVID-19 [4, 10]. Therefore, we suggest that all outpatients should be triaged before admission to reduce the possibility of exposure in hospital. In CG, to screen for suspected infections, patients were subjected to chest CT scans and new coronavirus nucleic acid tests before admission, which explains the longer waiting time before admission in CG. In addition, the provinces were relatively isolated during the pandemic, therefore, compared with PCG, the proportion of local patients in CG had increased.

After admission, patients were isolated in separate single-room wards without contact to surgeons or nurses. If the fever was lower than 37.3 °C or other symptoms associated with pneumonia were absent after 3 days of admission, surgical procedures would then be performed. The waiting time before surgery was, therefore, longer. During the pandemic, routine surgical techniques should be based on the principles of safety and efficiency, with the main purpose of reducing the incidences of postoperative complications while accelerating the patient’s recovery and discharge [13–15]. It was necessary to avoid performing surgical procedures beyond the established guidelines, including oversized lymph node dissections with uncertain effects and complex digestive tract reconstruction methods. For better surgical outcomes, attention should be paid during surgery to reduce the risk of bleeding. This decreases the chances for blood transfusion.

During COVID-19 period, more laparoscopic surgeries were performed. COVID-19 is mainly transmitted through respiratory droplets, but the risk of COVID-19 transmission is greatly increased during aerosol generation procedure (AGP) in laparoscopic surgery [16]. Compared with open surgery, there are concerns that the leaked CO_2_ and smoke may lead to the generation of COVID-19 contaminated aerosols, which may be due to the application of ultrasonic surgical instruments, low gas motility of pneumoperitoneum, and gas expulsion through trocars or ports [17]. Therefore, The Intercollegiate General Surgery Guidance on COVID-19 and The Society of Gastrointestinal and Endoscopic Surgeons (SAGES) initially highlighted the risk of aerosolization during laparoscopic surgery, although their updated guidance acknowledged a lack of evidence [18, 19].

However, both open surgery and laparoscopy could generate surgical smoke. When necessary preventive measures are taken, smoke control can be achieved in the closed cavity of laparoscopic surgery, while it cannot be properly controlled in open surgery. The key factors for safe control of smoke hazards are smoke evacuation completely purified by filters and intelligent use of ultrasonic surgical instruments.

We have rigorously analyzed the researches associated with surgical smoke and found there was lacking of enough evidence that laparoscopic surgery is routinely prohibited simply due to the aerosol generation procedure during operation. Moreover, there was less evidence that had shown relationship between COVID-19 transmission and surgical smoke generated by ultrasonic surgical instruments [20–23]. By the way, during the COVID-19 pandemic, we also use the smoke extractor with vacuum motors which were applied to inhale smoke from the surgical site through a completely enclosed vacuum tube and filter. Medical staffs were therefore protected from potential contamination. Therefore, we have used laparoscopy more frequently during COVID-19, and information about laparoscopic surgeries, such as surgery time, blood loss and complications, indicated that the method was safe and feasible. However, although we have not found any evidence of particular risk in laparoscopic surgery, the risk might still exist. Further investigation in this field is of critical importance.

The hospitalization costs were significantly increased in CG. As for the reason, we would like to elaborate on prolonged hospital stay. Patients in CG were observed in the separate single-room wards for 3 days to prevent potential infection. Therefore, the preoperative hospital stay was longer. As for the post-operative hospital stay, during the COVID-19 period, patients had to have their stitches removed in outpatient clinics and local hospitals after discharge. This increased the risk of unnecessary viral infections. It was better to stay longer in our department until stitches were removed. The hospital stay period after surgery was, therefore, longer in CG.

We found no statistically differences in postoperative fever. If the patient developed fever of unknown cause after surgery, appropriate ward isolation measures should be taken and measurements of postoperative blood routine, C-reactive protein, procalcitonin, chest CT, and new coronavirus nucleic acid tests were necessary.

### Limitations

This study had some limitations. Firstly, the presented results are for a short-term follow-up period which fails to illustrate the long-term outcomes such as progression-free survival and mortality. Secondly, oversized lymph node dissections with uncertain effects were not performed beyond authoritative guidelines, which may have also impact on oncologic outcome. More studies are needed to investigate the impact of these procedures on oncologic outcomes. Thirdly, the study was retrospectively performed in a single center and may therefore involve selection bias.

## Conclusions

In conclusion, there are no studies on the impact of COVID-19 on gastric cancer patients. The full impact of COVID-19 on surgical procedures is still unknown. As this pandemic has affected global economics, politics, hospital management, health strategies and personnel, its impact may only become evident in the long term. This study provides an opportunity for surgical residents to reflect on their own service and any contingency plans they have to tackle this crisis.

## Data Availability

The datasets used and/or analysed during the current study are available from the corresponding author on reasonable request.

## Abbreviations

COVID-19: Coronavirus disease 2019
PCG: Pre-COVID-19 group
CG: COVID-19 group
IQR: Interquartile range
WHO: World Health Organization
NCAT: Neoadjuvant chemotherapy
NCCN: National Comprehensive Cancer Network
AGP: Aerosol generation procedure
SAGES: Society of Gastrointestinal and Endoscopic Surgeons
